# Association of Cardiovascular Disease and Pancreatitis: What Came First, the Chicken or the Egg?

**DOI:** 10.3390/jcm12227101

**Published:** 2023-11-15

**Authors:** Bing Chen, Aleena Moin, Hafeez Ul Hassan Virk, Hani Jneid, Salim S. Virani, Chayakrit Krittanawong

**Affiliations:** 1 Department of Gastroenterology and Hepatology, Geisinger Medical Center, Danville, PA 17822, USA; 2 Department of Internal Medicine-Pediatrics, Geisinger Medical Center, Danville, PA 17822, USA; 3 Harrington Heart & Vascular Institute, Case Western Reserve University, University Hospitals Cleveland Medical Center, Cleveland, OH 44106, USA; 4 John Sealy Distinguished Centennial Chair in Cardiology, Division of Cardiology, University of Texas Medical Branch, Houston, TX 77058, USA; 5 Section of Cardiology and Cardiovascular Research, Department of Medicine, Baylor College of Medicine, Houston, TX 77030, USA; 6 Office of the Vice Provost (Research), The Aga Khan University, Karachi 74800, Pakistan; 7 Cardiology Division, NYU Langone Health and NYU School of Medicine, New York, NY 10016, USA

**Keywords:** acute pancreatitis, cardiovascular disease, chronic pancreatitis

## Abstract

(1) Background: Recent studies suggest an association between pancreatitis and cardiovascular disease. This article aims to review the available evidence linking cardiovascular disease with acute and chronic pancreatitis. (2) Methods: A comprehensive search was conducted on the PubMed/MEDLINE database from inception to April 2022 using Medical Subject Heading and keywords related to pancreatitis and cardiovascular disease. The search was limited to English-language literature involving human subjects, and various study types, including observational studies, case–control studies, cohort studies, and clinical trials, were screened for eligibility. Following data extraction, the authors conducted a narrative synthesis of the studies. (3) Results: Longitudinal studies indicate that a history of acute pancreatitis is associated with an increased risk of acute atherosclerotic cardiovascular disease and acute coronary syndrome. Elevated triglyceride levels (>2000 mg/dL) have a temporal relationship with acute pancreatitis. Cross-sectional studies have shown that acute pancreatitis is associated with cardiac injury during the acute phase. Based on longitudinal studies, chronic pancreatitis is associated with an increased risk of cerebrovascular diseases. However, data regarding the relationship between chronic pancreatitis and myocardial infarction are conflicting. (4) Conclusions: Based on the available evidence, having a history of acute pancreatitis appears to increase the risk of acute atherosclerotic cardiovascular disease. However, there is insufficient evidence to conclude whether chronic pancreatitis is associated with cardiovascular disease, and no definitive studies have yielded conflicting results.

## 1. Introduction

Acute pancreatitis is one of the most common gastrointestinal conditions that results in hospital admission in the United States [1]. It is an inflammatory disorder of the pancreas that occurs when damage to the acinar cells precipitates inappropriate release and activation of trypsinogen, which eventually leads to pancreatic parenchyma autodigestion [1]. This results in local injury, systematic inflammatory response syndrome, and, in severe cases, organ failure [2]. Acute pancreatitis is a potentially life-threatening condition with a case fatality rate of around 5% [2]. On the other hand, chronic pancreatitis is a chronic inflammatory condition of the pancreas that leads to fibrosis and scarring [3]. It is a progressive disease that can manifest in abdominal pain, malnutrition, and exocrine and endocrine insufficiency [3]. Acute and chronic pancreatitis are closely related, and studies have shown that 36% of patients with recurrent acute pancreatitis eventually develop chronic pancreatitis [4]. The global incidence of acute pancreatitis is estimated to be approximately 34 cases per 100,000 people in the general population, and that of chronic pancreatitis is estimated to be around 10 cases per 100,000 people [5]. Pancreatitis is a public health problem with an increasing burden, as evidenced by a study that showed increased age-standardized prevalence and years lived with disability rate of pancreatitis increased over the last three decades [6].

Atherosclerotic cardiovascular disease (ASCVD) refers to conditions that include acute coronary syndrome (ACS), myocardial infarction (MI), stable or unstable angina, coronary or other arterial revascularization, stroke, transient ischemic attack, or peripheral artery disease, which are all of atherosclerotic origin [7]. Cardiovascular diseases (CVDs) are a leading contributor to global morbidity and mortality, affecting more than 500 million people and resulting in 19 million deaths annually [8] (p. 369). Several studies have shown that certain gastrointestinal diseases, including inflammatory bowel disease, celiac disease, and gallstone disease, are associated with an increased risk of CVD, while the association between pancreatitis and CVD has been reported but remains to be fully explored [9,10,11]. For example, CVD has been linked to an increased risk of acute pancreatitis [12]. Acute pancreatitis, in turn, has been associated with cardiac injury, and increased risk of ASCVD [13,14]. Similarly, the relationship between chronic pancreatitis and myocardial infarction has also been investigated in different studies [15,16].

Given the potential interrelationship between pancreatitis and CVD, healthcare professionals need to be aware of the potential connection between these conditions to better optimize patient care. Therefore, this review aims to summarize the current evidence on the relationship between pancreatitis and CVD.

## 2. Methods

**Figure 1:**
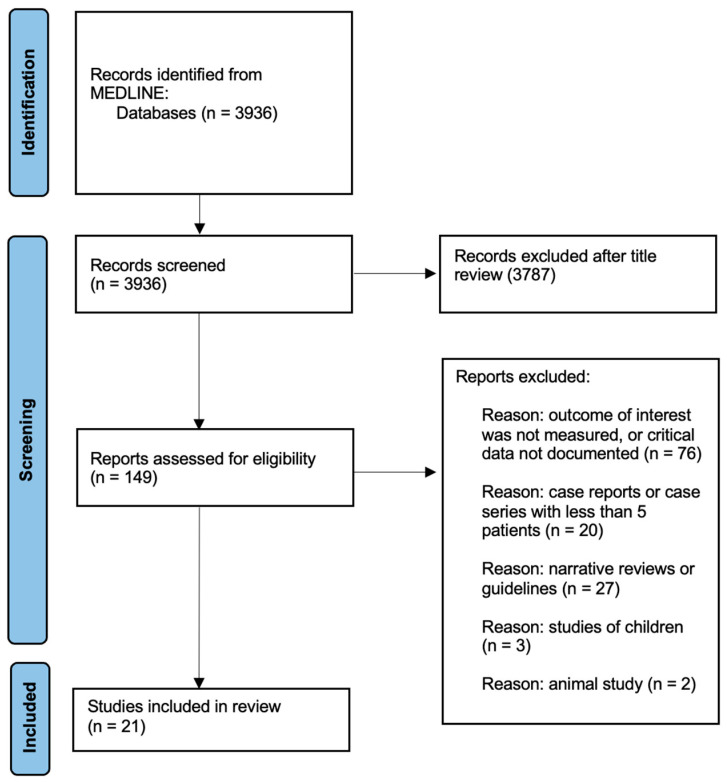
PRISMA flow diagram of study selection.

To identify relevant studies, a comprehensive search of the Pubmed/MEDLINE database was conducted to retrieve from inception to April 2022. A combination of Medical Subject Headings (MeSH) terms and text words related to pancreatitis and cardiovascular disease were used. MeSH terms included “pancreatitis”, “cardiovascular diseases”, “coronary artery disease”, “acute coronary syndrome”, “atherosclerosis”, “heart injuries”, “myocardial infarction”, and “heart failure”, along with additional keywords, such as “hypertriglyceridemia”. Only English-language literature involving human subjects was included, and a range of study types, including observational studies, case–control studies, cohort studies, and clinical trials, were eligible for inclusion. After screening and data extraction (Figure 1), the authors conducted a narrative synthesis of the studies, with the extracted data being summarized into tables for easy comparison and synthesis.

## 3. Results and Discussions

### 3.1. Acute Pancreatitis

The key studies on acute pancreatitis are listed in Table 1. 

#### 3.1.1. CVD and Risk of Acute Pancreatitis

Intima media thickness (IMT) serves as an important indicator of atherosclerosis and the subsequent development of CVD. In a cross-sectional study by Kurkco et al., the researchers evaluated the IMT of the common and internal carotid arteries among 102 patients hospitalized for acute pancreatitis [17]. The patients were categorized based on the severity of the pancreatitis according to the revised Atlanta scores. The results showed that patients with higher common and internal carotid IMT (>0.775 mm) had more severe acute pancreatitis compared to those with lower IMT (*p* = 0.000). Therefore, the findings suggest a potential association between increased IMT and the severity of acute pancreatitis.

Bexelius et al. conducted a cross-sectional study in Sweden to investigate the association between CVD and the risk of acute pancreatitis. The study included 6161 patients with first-time acute pancreatitis who were compared against 61,637 matched controls [12]. After adjusting for various confounding factors such as education, alcohol disease, gallstone disease, chronic obstructive pulmonary disease (COPD), type 2 diabetes, several distinct medications, and other cardiovascular disorders, the authors found that the presence of any cardiovascular disorder was associated with an increased risk of acute pancreatitis with an adjusted odds ratio (OR) of 1.35 (95% CI: 1.25–1.45). Specifically, hypertension was seen in 22% of cases vs. 12% of controls (adjusted OR 1.34, 95% CI: 1.24–1.46), while ischemic heart disease was seen in 16% of cases vs. 10% of controls (adjusted OR 1.10, 95% CI: 1.01–1.20). No such association was noted for congestive heart disease or stroke. Overall, the study indicated that CVD is associated with an increased risk of pancreatitis. Although this association could be confounded by secondary outcomes such as tobacco use or metabolic disease, the study did attempt to control for type 2 diabetes and COPD (due to lack of explicit tobacco smoking data) and did not find a substantial change in the odd ratio, favoring an independent relationship between CVD and acute pancreatitis. It is important to note that given the cross-sectional nature of this study, the temporal association between acute pancreatitis and ischemic heart disease cannot be ascertained.

#### 3.1.2. Risk of Atherosclerosis and Coronary Artery Disease (CAD) in Patients with Acute Pancreatitis

Sung et al. conducted a longitudinal analysis using Taiwan’s National Health Insurance database, which included 2607 patients newly diagnosed with acute pancreatitis from 2000 to 2008, along with 10,948 persons without a history of pancreatitis as the control group [18]. Both cohorts had no prior history of acute ASCVD, which was defined as acute myocardial infarction and stroke, and were followed up from the index date until 31 December 2013, or until death. The authors found that a history of acute pancreatitis was associated with an increased risk of acute ASCVD with an adjusted hazard ratio (HR) of 1.76 (95% CI: 1.47–2.12) when compared to the control cohort.

#### 3.1.3. Hypertriglyceridemia and Acute Pancreatitis

Adiamah et al. performed a systematic review in 2018, which included 38 studies [19]. The review showed that the reported proportion of hyperlipidemic pancreatitis in patients with acute pancreatitis ranged between 2.3% and 53%. The median admission triglyceride concentration was 3785 mg/dL (range 1205–9612 mg/dL).

Amblee et al. performed a retrospective cross-sectional study spanning a decade (from 2003 to 2013) and studied 1157 adults with serum triglyceride levels > 1000 mg/dL [20]. They found that the prevalence of acute pancreatitis was 9.2% in this population. Baseline characteristics showed that patients with hypertriglyceridemic acute pancreatitis were younger (41.3 vs. 50 years; *p* < 0.001) compared to patients without hypertriglyceridemic acute pancreatitis. Furthermore, triglyceride levels > 2000 mg/dL were associated with a higher incidence of acute pancreatitis (22% vs. 5%). Excessive alcohol intake (OR 3.9, 95% CI: 2.5–6.0), gallstone disease (OR 3.9, 95% CI: 1.4–10.8), and triglycerides levels > 2000 mg/dL (OR 4.8, 95% CI: 3.1–7.4) were all independently associated with acute pancreatitis.

A longitudinal study by Copeland et al. attempted to observe the association of hypertriglyceridemia with acute pancreatitis among patients admitted to the Veterans Affairs hospital in the USA from 2006 to 2009 [21]. The mean triglyceride levels were recorded monthly until admission for acute pancreatitis. Ultimately, a small positive association was found between elevated triglycerides (>2000 mg/dL) and days from triglyceride assay to acute pancreatitis admission with an adjusted hazard ratio (HR) of 1.38 (95% CI: 1.16–1.63). However, no association was noted with lower levels of triglycerides, which the study described as 200 mg/dL to 1999 mg/dL.

In summary, hyperlipidemic pancreatitis is a common type of acute pancreatitis and a temporal relationship between elevated triglycerides (>2000 mg/dL) and the risk of acute pancreatitis is suggested.

#### 3.1.4. Congestive Heart Failure (CHF) and Acute Pancreatitis

The authors did not find any clinical studies investigating the relationship between acute pancreatitis and CHF. However, a cross-sectional study using the National Inpatient Sample demonstrated that chronic heart failure was associated with increased rates of respiratory failure (9.57% vs. 2.34%, *p* < 0.001), intubation (4.90% vs. 1.56%, *p* < 0.001), in-hospital mortality (3.16% vs. 0.67%, *p* < 0.001), and longer length of stay (6.75 days vs. 4.67 days) in patients admitted with a primary diagnosis of acute pancreatitis [22].

#### 3.1.5. Acute Pancreatitis-Associated Cardiac Injury

A cross-sectional study in Mexico included 28 patients, with 55% having mild acute pancreatitis and 45% having moderate/severe acute pancreatitis [13]. The authors found that 67% had increased pro-brain natriuretic peptide levels, 52% had abnormal electrocardiogram (ECG) findings (with at least one of the following ECG findings: sinus tachycardia, bradycardia, QRS prolongation, QTc prolongation, right bundle branch block, left anterior fascicular branch block, non-specific repolarization changes, T-wave inversion, ST depression, ST elevation, left atrium abnormality, atrial fibrillation, inferior QS complex), 48% had abnormal echocardiographic findings (with at least one of the following findings on echocardiogram: right ventricle dilation, diastolic dysfunction, pulmonary hypertension, tricuspid regurgitation, mitral regurgitation, aortic insufficiency, left atrial dilation, hypokinesis, pulmonary insufficiency, systemic ejection fraction), and 18% had increased troponin levels. Compared to patients with mild pancreatitis, patients with moderate/severe acute pancreatitis did not show any difference in the incidence of abnormal cardiovascular findings (ACFs: defined as any abnormalities of ECG findings, echocardiographic findings, troponin level, and pro-brain natriuretic peptide levels). Nineteen patients (70%) had follow-up testing, and most of the initial ACFs did not persist on repeated follow-up testing. Thandassery et al. performed transthoracic echocardiography on 72 patients with severe acute pancreatitis and hypotension [23]. They found cardiovascular dysfunction in 65% of patients, including 60% with diastolic dysfunction, 17% with systolic dysfunction, and 23% with combined dysfunction.

A comprehensive review by Khan et al. examined 34 cases of acute pancreatitis that presented with electrocardiographic changes suggesting acute myocardial infarction and found elevated troponin levels in 54% of cases [25]. Of the 23 patients who underwent cardiac echocardiography, 48% had wall motion abnormalities, and 4 of them had Takotsubo cardiomyopathy. Only 1 patient, out of 17 who underwent coronary angiography, had a thrombotic lesion and occlusion of the coronary artery. T wave changes and ST Segment depression are the most common changes in acute pancreatitis, observed in up to 50 percent of patients, and most ECG changes resolve following treatment [26]. Proposed mechanisms for ECG changes in acute pancreatitis include metabolic disturbances, direct injury to the pericardium or myocyte membrane, coagulopathy, coronary artery spasm, and hemodynamic instability [26].

In summary, cross-sectional studies have shown that during the acute phase of acute pancreatitis, patients frequently exhibit abnormal cardiovascular findings, including increased levels of pro-brain natriuretic peptide and troponin, as well as abnormal ECG and echocardiographic findings, all of which indicate cardiac injury.

#### 3.1.6. Acute MI and Acute Pancreatitis

Chung et al. conducted a longitudinal study using the Taiwan National Health Insurance Research Database [14]. They assessed 87,068 patients who were newly diagnosed with acute pancreatitis from 2000 to 2010, aged >20 years, and had no history of ACS. They also evaluated 348,272 matched participants in the control cohort. These patients were followed from the index date, defined as the first admission date for acute pancreatitis, until a diagnosis of ACS was confirmed, the patient was censored for loss at follow-up, death, withdrawal from the insurance system, or until 31 December 2011. The incidence of ACS was 5.44 vs. 3.03 per 1000 person-years in the acute pancreatitis cohort vs. the control cohort, with an adjusted HR of 1.24 (95% CI: 1.19–1.30) after adjustment for sex, age, and comorbidities, including hyperlipidemia. They conducted a subgroup analysis on idiopathic acute pancreatitis, excluding cases related to biliary, alcohol, and hypertriglyceridemia. This analysis revealed that idiopathic acute pancreatitis was associated with an increased risk of ACS with an adjusted HR of 1.35 (95% CI: 1.28–1.43). The longitudinal study from Korea by Jang et al. enrolled a total of 2,746,988 participants with type 2 diabetes mellitus who underwent a general health examination between 2009 and 2012, of which 3810 had a history of acute pancreatitis, and the researchers followed them through 2018 [24]. The study showed that a history of acute pancreatitis was associated with an increased risk of MI and mortality, with an adjusted HR of 1.998 (95% CI: 1.733–2.303) and 2.353 (95% CI: 2.200–2.215), respectively after adjustment for age, sex, and comorbidities, including hyperlipidemia. In summary, longitudinal studies indicate an association between acute pancreatitis and increased risk of ACS and MI.

#### 3.1.7. Potential Mechanisms between Acute Pancreatitis and ASCVD

Atherosclerosis is the leading cause of CVD. It involves chronic inflammation of the arterial vessel walls, driven by an interplay between maladaptive immune responses and metabolic imbalances, including a hyperlipidemic environment [27,28,29]. The release of inflammatory cytokines, such as interleukin (IL)-1β, IL-6, IL-8, IL-10, IL-18, and tumor necrosis factor-α, occurs in acute pancreatitis [30,31]. These cytokines can induce systemic inflammatory response, which may trigger endothelial dysfunction and lead to the rupture of coronary atherosclerotic plaques, causing CVD in the short term [32,33]. They are associated with the pathogenesis of atherosclerosis, leading to CVD in the long term [34].

Another potential link between acute pancreatitis and CVD is infection. A UK Biobank prospective cohort study showed that hospitalization for an infection, largely irrespective of the infection type, increased the risk of major cardiovascular events, defined as MI, cardiac death, or fatal or nonfatal stroke, with a HR of 7.87 (95% CI: 6.36–9.73) during the first month after infection, and 1.47 (95% CI: 1.40–1.54) during the mean follow up of 11.6 years after adjustment for traditional cardiovascular risk factors [35]. Previous studies have shown that microorganisms and their remnants, complexed with homocysteinylated low-density lipoprotein, may play a causal role in the pathogenesis of atherosclerosis and the formation of vulnerable plaque [36,37]. Infection is a common complication in acute pancreatitis and is associated with increased mortality [38,39]. Extra-pancreatic infectious complications occur in 32% of patients with acute pancreatitis [40]. Necrotizing pancreatitis occurs in approximately 20% of patients with acute pancreatitis, and it is associated with 40–70% of pancreatic infection [41,42]. Based on these data, one might extrapolate that infections related to acute pancreatitis increase the risk of CVD.

**Figure 2:**
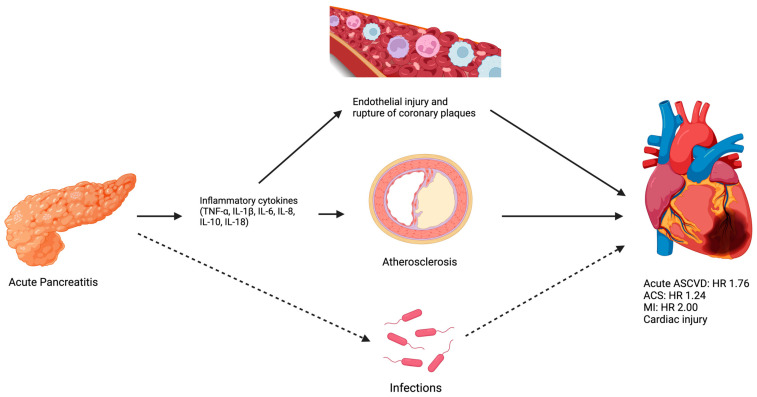
Acute pancreatitis is associated with an elevated risk of acute ASCVD, ACS, MI, and cardiac injury. Potential mechanisms involve the release of inflammatory cytokines, which can lead to endothelial injury and the rupture of coronary atherosclerotic plaques. The inflammatory cytokines are also associated with the pathogenesis of atherosclerosis. Additionally, infections are a common complication of acute pancreatitis and have been found to be associated with major cardiovascular events. ASCVD = atherosclerotic cardiovascular disease; ACS = acute coronary syndrome; MI = myocardial infarction; HR = hazard ratio.

The mechanisms and relationship between acute pancreatitis and ASCVD are illustrated in Figure 2.

### 3.2. Chronic Pancreatitis

The key studies on chronic pancreatitis are listed in Table 2. 

#### 3.2.1. CVD and Risk of Chronic Pancreatitis

No clinical studies studying the risk of chronic pancreatitis in patients with CVD were found in our search.

#### 3.2.2. Risk of Atherosclerosis and CAD in Patients with Chronic Pancreatitis

Gullo et al. conducted a cross-sectional study investigating cardiovascular lesions in 54 patients with proven chronic pancreatitis against 54 controls [43]. The study identified 8 patients and 3 controls with electrocardiographic changes that indicated coronary artery disease, including unequivocal Q wave alternations and resting ECG changes compatible with myocardial ischemia. A cross-sectional study by Lee et al. reviewed panoramic images of 32 men with alcohol-related chronic pancreatitis resulting in type 3c diabetes mellitus (ARCP-DM) and found that 8 (25%) had calcified carotid artery atheromas, defined as radiopaque lesions in a verticolinear orientation relative to the hyoid bone or parallel to the cervical vertebrae in the region of the intervertebral space between C3 and C4 [44]. This association between ARCP-DM and the presence of atheroma on the panoramic image was statistically significant (*p* < 0.05) compared to the historical general-population cohort with a 3% rate.

In two separate longitudinal studies, the association between chronic pancreatitis and cerebrovascular disease was investigated. Bang et al. used Danish registries to analyze 11,972 cases of chronic pancreatitis (71,814 person-years) and 119,720 controls (917,436 person-years) [16]. They found that chronic pancreatitis was associated with a higher risk of cerebrovascular disease, with an adjusted HR of 1.3 (95% CI: 1.2–1.4). Wong et al. conducted a similar study, using the Taiwan National Health Insurance Program, which included 16,672 patients with chronic pancreatitis (80,207 person-years) and 65,877 controls (365,209 person-years) [45]. Their study also demonstrated an association between chronic pancreatitis and cerebrovascular disease, with an adjusted HR of 1.27 (95% CI 1.19–1.36) after adjusting for sex, age, and baseline comorbidities. In summary, longitudinal studies have shown a significant association between chronic pancreatitis and cerebrovascular disease.

#### 3.2.3. Hypertriglyceridemia and Chronic Pancreatitis

No clinical studies have been found in our search to study the relationship between hypertriglyceridemia and chronic pancreatitis.

#### 3.2.4. CHF and Chronic Pancreatitis

Pancreatic exocrine insufficiency (PEI), which is characterized by insufficient secretion of pancreatic enzymes and/or sodium bicarbonate, is one of the most common complications of chronic pancreatitis [51]. In a longitudinal study including 430 patients with a mean age of 47.8 years, PEI was present in 29.3% of patients with chronic pancreatitis [46]. Xia et al. conducted a study on 104 hospitalized patients with CHF and found that 56.7% had PEI, indicated by fecal elastase-1 levels, compared to 0.00% in the control group (*n* = 20) [47]. Fecal elastase-1 levels were also found to decrease with an increasing severity of CHF. In another cross-sectional study by Miroslav et al., PEI was found in 6.9% of patients with CHF, including 3.45% of patients with severe PEI and 3.45% with mild PEI [48]. Ozcan et al. found a significant difference in fecal elastase levels between patients with severe acute decompensated heart failure (HF) (50% with severe PEI and 20% with moderate PEI) vs. the control and mild acute decompensated HF group (around two-thirds had normal pancreatic function) [49]. Dam et al. proposed that the acceleration of the development of PEI was related to deranged hemodynamics (e.g., congestion and hypoperfusion), chronic inflammation, and autonomic dysfunction of the pancreas [52]. On the contrary, PEI can aggravate the wasting of advanced HF and may result in micronutrient deficiencies, further deteriorating heart failure [52].

In summary, cross-sectional studies suggest an association between CHF or severe acute decompensated HF and PEI. However, longitudinal studies are needed to confirm this association.

#### 3.2.5. Chronic Pancreatitis-Associated Cardiac Injury

No clinical studies have been found in our search about chronic pancreatitis-associated cardiac injury.

#### 3.2.6. Acute MI and Chronic Pancreatitis

Hsu et al. conducted a retrospective longitudinal study by randomly selecting 17,405 patients with chronic pancreatitis and 69,620 matched controls from 2000 to 2010 [50]. During the follow-up period of 4.85 and 6.00 years for the two groups, respectively, the incidence of acute coronary syndrome was 4.89 vs. 2.28 per 10,000 person-years in the chronic pancreatitis group compared to the control group, with an adjusted HR of 1.40 (95% CI: 1.20–1.64), after adjusting for age, gender, and comorbidities of hypertension, diabetes, hyperlipidemia, cerebrovascular accident (CVA), atrial fibrillation, HF, COPD, chronic kidney disease, and acute pancreatitis. The study also revealed that patients with chronic pancreatitis aged ≤39 years exhibited the highest risk of ACS with an adjusted HR of 2.14 (95% CI: 1.13–4.02) compared to individuals without chronic pancreatitis. Compared to patients without chronic pancreatitis and lacking comorbidities, more comorbidities in patients with chronic pancreatitis were also predisposed patients to a higher risk of ACS with an adjusted HR of 9.67 (95% CI: 7.08–13.2) in patients with 5 or more comorbidities, followed by patients with 4, 3, 2 and 1 comorbidities, respectively. A cross-sectional study by Khan et al. included a total of 28,842,210 patients using the data from a commercial database [15]. They found that the overall prevalence of MI was 14.22% in the chronic pancreatitis group compared with 3.23% in the control group, with an adjusted OR of 1.453 (95% CI: 1.418–1.488, *p* < 0.0001) after adjusting for age, sex, race, and common risk factors for MI.

However, another longitudinal study using Danish registries from 1995 through to 2010 compared 11,972 patients with chronic pancreatitis and 119,720 sex-matched controls [16]. They did not find a significant association between chronic pancreatitis and myocardial infarction, with an HR of 0.9 (95% CI: 0.8–1.0) after adjusting for socioeconomic status, diabetes, and chronic pulmonary disease. In addition, the study by Sung et al. did not find a significant association between chronic pancreatitis and acute myocardial infarction, but this study was limited due to a low sample size of only 71 patients and 614 person-years follow-up [18].

In summary, the longitudinal studies investigating the association between chronic pancreatitis and acute coronary syndrome, or myocardial infarction have yielded conflicting results, making it challenging to draw a definitive conclusion.

## 4. Conclusions

Our review is aimed at increasing awareness of the association between pancreatitis and cardiovascular diseases. It has shown that, according to longitudinal studies, there is an association between a history of acute pancreatitis and an elevated risk of acute atherosclerotic cardiovascular disease and acute coronary syndrome. Cross-sectional studies indicate that acute pancreatitis is related to cardiac injury during the acute phase. However, the evidence regarding the relationship between chronic pancreatitis and cardiovascular disease is inconclusive, and conflicting results have been reported. Further research is necessary to develop risk stratification to identify individuals with acute pancreatitis who have the highest risk of developing cardiovascular diseases and to investigate the potential for early intervention in cardiovascular risk factors in this population.

## Figures and Tables

**Table 1:** Summary of key studies of acute pancreatitis.

Reference (First Author, Year of Publication, Country)	Type of Study (Longitudinal vs. Cross-Sectional)	Patients, *n*	Mean Age (Years)	Outcomes
K Kurkcu et al., 2018, Turkey [17]	Cross-sectional	101	50	Common and internal carotid artery intima-media thickness above > 0.755 were associated with more severe AP. (*p* = 0.000).
Bexelius et al., 2013, Sweden [12]	Cross-sectional	6161 AP 61,637 controls	N/A	CVD was positively associated with risk of AP (adjusted OR 1.35, 95% CI: 1.25–1.45).
Sung et al., 2021, Taiwan [18]	Longitudinal	2607	N/A	The adjusted HR of acute ASCVD was 1.76 (95% CI 1.47–2.12) for people with AP.
Adiamah et al., 2018, UK [19]	Systematic review with 38 studies	1979	NA	The reported proportion of hyperlipidemic pancreatitis ranged between 2.3 and 53% in patients with AP.
Amblee et al., 2018, USA [20]	Cross-sectional	1157	49.2	The prevalence of AP was 9.2% in patients with a serum triglyceride level ≥ 1000 mg/dL.
Copeland et al., 2018, USA [21]	Longitudinal	20,608	60.4	Elevated triglycerides (>2000 mg/dL) levels were positively associated with days to acute pancreatitis admissions from triglyceride assessment (HR 1.38, CI 95: 1.16–1.63).
Mehta et al., 2019, USA [22]	Cross-sectional	1,356,659 AP with 69,657 CHF	69.7 without CHF vs. 51.1 with CHF	CHF is associated with more respiratory failure, intubation, higher in-hospital mortality, and longer length of stay in patients admitted for AP.
Chacon-Portillo et al., 2017, Mexico [13]	Cross-sectional study	27	48	67% with increased pro-brain natriuretic peptide levels, 52% had abnormal ECG findings, 48% had abnormal echo findings, and 18% had increased troponin levels in the acute phase of AP.
Thandassery et al. 2017, India [23]	Cross-sectional	72	41 (median age)	In patients with severe pancreatitis and hypotension, 60% with diastolic dysfunction, 17% with systolic dysfunction, and 23% with combined dysfunction on ECHO.
Chung et al., 2017, Taiwan [14]	Longitudinal	87,068 with AP, 348,272 controls	53	Incidence of ACS in AP vs. control: 5.44 vs. 3.03 per person-years, with adjusted HR of 1.24 (95% CI: 1.19–1.30).
Jang et al., 2022, Korea [24]	Longitudinal	3810 with AP, 2,258,910 control	55.0 with AP vs. 55.7 in control	AP is associated with increased risk for MI with an adjusted HR of 1.998 (95% CI 1.733–2.303) in patients with diabetes.

AP = acute pancreatitis; CVD = cardiovascular diseases; ASCVD = atherosclerotic cardiovascular diseases; OR = odds ratio; HR = hazard ratio; CI = confidence interval; CHF = congestive heart failure; ECG = electrocardiogram; ECHO = echocardiography; ACS = acute coronary syndrome; MI = myocardial infarction.

**Table 2:** Summary of key studies of chronic pancreatitis.

Reference (First Author, Year of Study, Country)	Type of Study (Longitudinal vs. Cross-Sectional)	Patients, *n*	Mean Age	Outcomes
Gullo et al., 1982, Italy [43]	Cross-sectional	54 CP, 54 controls	N/A	Arterial involvement in 18 patients vs. 5 controls (33% vs. 9%, *p* < 0.01); ECG changes indicating CAD in 8 patients vs. 3 controls.
Lee et al., 2018, USA [44]	Cross-sectional	32	61.7	Significant association between a diagnosis of ARCP-DM and presence of atheroma on the panoramic image: 25% vs. 3% (*p* < 0.05) in patients with alcohol-related chronic pancreatitis vs. general population.
Bang et al., 2014, Denmark [16]	Longitudinal	11,972 CP, 11,972 controls	54.5	CP is associated with higher prevalence of cerebrovascular disease with an adjusted HR of 1.3 (95% CI: 1.2–1.4), but not MI with an adjusted HR of 0.9 (95% CI: 0.8–1.0).
Wong et al., 2016, Taiwan [45]	Longitudinal	16,672 CP, 65,877 controls	47.5	Incidence of cerebrovascular disease was 14.2 vs. 11.5 per 1000 person-years with an adjusted HR of 1.27 (95% CI: 1.19–1.36).
De la Iglesia et al., 2018, Spain [46]	Longitudinal	430	47.8	PEI was present in 29.3% of patients with chronic pancreatitis
Xia et al., 2017, China [47]	Cross-sectional	104	70.4	The prevalence of PEI (*n* = 59) is 56.7% in patients with CHF, compared to 0.00% in the control group.
Miroslav et al., 2015, Sweden [48]	Cross-sectional	87	74.7	PEI were diagnosed in 6.9% of patients with CHF: severe PEI 3.45%, and mild PEI 3.45%.
Ozcan et al., 2015, Istanbul [49]	Cross-sectional	52	67.5	In the severe acute decompensated HF group, 20% had mild to moderate PEI, and 50% had severe PEI. In the mild acute decompensated HF group, 9.4% had mild to moderate PEI, and 12.5% had severe PEI. In the control group, 12.9% had mild to moderate PEI, and 19.4% had severe PEI.
Hsu et al., 2016, Taiwan [50]	Longitudinal	17,405 CP, 69,620 controls	48.3	ACS incidence 4.89 vs. 2.28 per 10,000 person-years in CP vs. controls with an adjusted hazard ratio of 1.40 (95% CI: 1.20–1.64).
Khan et al., 2021, USA [15]	Cross-sectional	63,230 CP, 28,778,980 controls	N/A	Prevalence of MI 14.22% vs. 3.23% in CP vs. controls (*p* < 0.0001), with an adjusted OR 1.453 (95% CI: 1.418–1.488).
Sung et al., 2021, Taiwan [18]	Longitudinal	71 CP	N/A	The adjusted HR of acute ASCVD was 3.42 (95% CI: 1.69–6.94) in CP vs. controls.

ECG = electrocardiogram; CAD = coronary artery disease; CP = chronic pancreatitis; ARCP-DM = alcohol-related chronic pancreatitis resulting in type 3c diabetes mellitus; HR = hazard ratio; CI = confidence interval; PEI = pancreatic exocrine insufficiency; CHF = congestive heart failure; HF = heart failure; ACS = acute coronary syndrome; MI = myocardial infarction; OR = odds ratio; ASCVD = atherosclerotic cardiovascular disease.

## Data Availability

No new data were created.
